# The NF-ĸB p50 subunit generated by KPC1-mediated ubiquitination and limited proteasomal processing, suppresses tumor growth

**DOI:** 10.1186/s12935-023-02919-5

**Published:** 2023-04-13

**Authors:** Yelena Kravtsova-Ivantsiv, Gilad Goldhirsh, Ciprian Tomuleasa, Eli Pikarsky, Aaron Ciechanover

**Affiliations:** 1 The Rappaport Faculty of Medicine and Research Institute and the Rappaport Technion Integrated Cancer Center (R-TICC), Technion-Israel Institute of Technology, P.O. Box 9649, 3109601 Haifa, Israel; 2 Department of Hematology-Medfuture Research Center for Advanced Medicine, Iuliu Hatieganu University of Medicine and Pharmacy, Cluj-Napoca, Romania; 3 The Lautenberg Center for Immunology and Cancer Research, Institute for Medical Research Israel-Canada (IMRIC), Hebrew University–Hadassah Medical School, 9112000 Jerusalem, Israel

**Keywords:** NF-ĸB p50, Tumor-suppression, PDL1, KPC1, PROTAC, Chemokines, Ubiquitin ligase, Proteasome

## Abstract

Nuclear factor-ĸB (NF-ĸB) is an important transcriptional regulator of key cellular processes, including cell cycle, immune response, and malignant transformation. We found that the ubiquitin ligase Kip1 ubiquitination-promoting complex subunit 1 (KPC1; also known as Ring finger protein 123 – RNF123) stimulates ubiquitination and limited proteasomal processing of the p105 NF-ĸB precursor to generate p50, the active subunit of the heterodimeric transcription factor. KPC1 binds to the ankyrin repeats’ (AR) domain of NF-ĸB p105 via a short binding site of 7 amino acids—968-WILVRLW-974. Though mature NF-ĸB is overexpressed and constitutively active in different tumors, we found that overexpression of the p50 subunit, exerts a strong tumor suppressive effect. Furthermore, excess of KPC1 that stimulates generation of p50 from the p105 precursor, also results in a similar effect. Analysis of transcripts of glioblastoma and breast tumors revealed that excess of p50 stimulates expression of many NF-ĸB-regulated tumor suppressive genes. Using human xenograft tumor models in different immune compromised mice, we demonstrated that the immune system plays a significant role in the tumor suppressive activity of p50:p50 homodimer stimulating the expression of the pro-inflammatory cytokines CCL3, CCL4, and CCL5 in both cultured cells and in the xenografts. Expression of these cytokines leads to recruitment of macrophages and NK cells, which restrict tumor growth. Finally, p50 inhibits the expression of the programmed cell death-ligand 1 (PDL1), establishing an additional level of a strong tumor suppressive response mediated by the immune system.

## Background

The ubiquitin–proteasome system (UPS) regulates a broad array of cellular processes (e.g., apoptosis, cell cycle, chromatin remodeling, immune response, and protein quality control) by covalent attachment of ubiquitin (Ub) moiety(ies) to different target substrates. The ‘canonical’ protein targeting signal for proteasomal recognition and degradation is a polyubiquitin-based chain, assembled through isopeptide bond between Lys48 of the proximal Ub moiety and the C-terminal Gly residue of the distal moiety. The chain is typically via the C-terminal Gly of the first Ub link of the chain to an internal Lys residue of the substrate. Accumulated information shows that a broad range of ubiquitin-based modifications such as monoubiquitination, multiple monoubiquitinations, linear chains, or ubiquitin chains branched via other than Lys48 linkages, can also result in proteasomal degradation, but in addition serve non-proteolytic functions such as modulation of signaling pathways or targeting of proteins to their appropriate subcellular destination [1–5].

The UPS is a main regulator of NF-ĸB [5–7]. Mature NF-ĸB is typically a heterodimer which consists of RelA (p65), RelB, or c-Rel, along with either NF-ĸB1 (p105/p50) or NF-ĸB2 (p100/p52) [6–8]. The variety of NF-ĸB combinations regulate numerous cellular processes, such as the immune response, cell death, proliferation and differentiation [9, 10]. The Rel proteins harbor a transactivation domain, which is lacking in p50 and p52. Thus, homodimers of p50 or p52 without a Rel protein partner, cannot activate transcription on their own, and rely on complex formation with transcriptional activators such as Bcl-3, IĸBζ, or HDACs [8].

Migration of the NF-ĸB dimers to the nucleus is inhibited by inhibitors of κB (IĸB) proteins which mask their nuclear localization signal (NLS) [11]. Following activation cues such as oxidative stress, bacterial and viral infections, cytokines and DNA damage, IĸB kinase (IKK) phosphorylates the IĸBs on specific serine residues. This phosphorylation triggers their ubiquitination and degradation by the proteasome. Consequently, the released dimers with exposed NLS migrate to the nucleus and initiate their specific transcriptional programs [12, 13]. Another essential role of the UPS in regulating the NF-ĸB activity is processing of the precursor proteins p100 and p105 to the active subunits p52 and p50, respectively [6, 7]. We demonstrated that proteasomal processing of p105 to p50 can be stimulated by multiple mono-ubiquitinations of the precursor protein [14].

Multiple studies found that NF-ĸB activity is upregulated in many malignancies [15]. For example, enhanced intensity of p65 staining in prostate adenocarcinoma was correlated with increased tumor grade, and hence aggressiveness [16]. In addition, ovarian carcinoma and borderline ovarian tumors exhibit higher expression of p50 and p65 compared to benign ovarian tumors [17]. The mechanisms that underlie the pro-tumorigenic activity of NF-κB are both direct and indirect. The indirect mechanisms include, among others, stimulation of expression of pro-inflammatory cytokines, that when secreted in an uncontrolled manner, stimulate malignant transformation. The direct mechanisms include stimulation of expression of growth-promoting factors (such as the Epidermal Growth Factor and its cognate receptors), and cell division mediators (such as cyclins). However, it appears that NF-ĸB can possess dual characteristics—both oncogenic and tumor suppressive [18]. For instance, in pancreatic cancer cells, IKKα downregulation alters NF-ĸB dimers composition leading to suppressed expression of Skp2—a part of the Ub ligase complex which targets p27^Kip^, a cell cycle inhibitor [19]. As a result, the inhibitor accumulates.

It is well established that immune checkpoints are crucial in modulating the immune response [20, 21]. Thus, the PD-1/PD-L1 axis is an important player that inhibits anti-tumoral activity of T cells and macrophages [22]. Accordingly, blocking the PD-1/PD-L1 axis triggers a potent anti-tumoral activity of natural killer (NK) cells [23]. In that context NF-ĸB was shown to stimulate expression of immune checkpoint molecules on the surface of tumor/immune cells [24]. RelA-based NF-ĸB dimers were shown to upregulate the deubiquitinating enzyme CSN5. Upregulation of CSN5 leads to the stabilization of membrane-anchored PD-L1, thus inhibiting anti-tumoral immune response and suppression of tumor cells [25]. The involvement of NF-ĸB in the expression of PD-L1 was also described in triple negative breast cancer (TNBC). PD-L1 and the transmembrane glycoprotein mucin1 (MUC1) are upregulated in TNBC, and play an important role in the aggressive behavior of this tumor. Mechanistic studies demonstrated that MUC1 stimulates PD-L1 transcription by recruiting MYC and NF-κB p65 to the PD-L1 promoter. Accordingly genetic or pharmacological targeting of MUC1, downregulates PD-L1 expression in TNBC cells [26].

In our study we found that the expression of PD-L1 is suppressed by either p50 or KPC1, resulting in an additional mechanism explaining the tumor suppressive activity of these proteins (see below).

## Main text

Activation of the NF-ĸB transcription factor is a multi-step process that involves the UPS all along [27, 28]. One of the unknown links in the chain was the identity of the ubiquitin ligase that catalyzes ubiquitination of p105 which is followed by limited proteasomal processing of NF-ĸB1 p105 to the active subunit p50. Applying a biochemical approach [29], we sequentially fractionated rabbit reticulocyte lysate using different chromatographic methods, and tested the resolved fractions for ubiquitination of labeled p105 in a cell free system. Mass spectrometric analysis of the active fractions from different chromatographical separations identified 58 peptides of KPC1 and 7 peptides of KPC2 [29]. KPC1 along with KPC2 is a heterodimeric ubiquitin ligase complex, that ubiquitinates the cyclin-dependent kinase inhibitor p27^KIP^, targeting it for proteasomal degradation [30]. In the heterodimer, KPC1 is the enzymatically active subunit, whereas KPC2 lacks catalytic activity, but is essential for the stability of KPC1 [31].

We confirmed that KPC1 directly recognizes and ubiquitinates p105 by establishing a cell free ubiquitination assay using purified KPC1 and [^35^S]methionine-labeled p105. We demonstrated a direct interaction between KPC1 and p105 also in cells, in a co-immunoprecipitation assay. This interaction results in ubiquitination of p105 which also depends on the presence of the ligase**.** Following silencing of KPC1 using small interfering RNA (siRNA) in HEK 293 cells, we found that KPC1 is responsible for basal processing of p105 to p50. It should be noted that signal-induced processing stimulated by expression of constitutively active IKKβS176,180E, also depends on the activity of KPC1 [29].

We found that KPC1 interacts with p105 via its C-terminal ARs domain. Moreover, when we removed all but one AR, we found that the single remaining AR is sufficient for the interaction [29]. To identify the p105-binding domain in KPC1, we deleted systematically stretches of amino acids in KPC1, covering the entire reading frame, and examined the interaction with p105 in a co-immunoprecipitation assay. We found that a short domain consisting of 7 amino acids—968-WILVRLW-974—mediates the interaction between two proteins, which is completely abrogated following its deletion [32]. Interestingly, a chimera protein consisting of the 7 amino acids attached to a short segment of KPC1 which contains the RING (Really Interesting New Gene) domain required for binding of the ubiquitin-conjugating enzyme, E2 (and therefore is essential for the ubiquitin-ligating activity of the protein), was able to interact with and to ubiquitinate p105. This activity of the mini-KPC1 was dependent on the presence of WILVRLW [32].

NF-ĸB is a transcription factor that controls expression of numerous genes that regulate normal homeostasis and are dysregulated in tumors. Thus, we decided to examine whether the tumorigenicity of human malignant cell lines is affected by overexpressed KPC1 or p50. Unpredictably, we found that overexpression of either KPC1 or p50, suppressed anchorage-independent growth of glioblastoma U87-MG, breast MDA-MB 231, and sarcoma U2OS cells compared to control cells. Not surprisingly, the tumor suppressive effect was strongly dependent on the RING domain of KPC1 and on the presence of p105 that is the precursor of p50 [29].

**Fig. 1:**
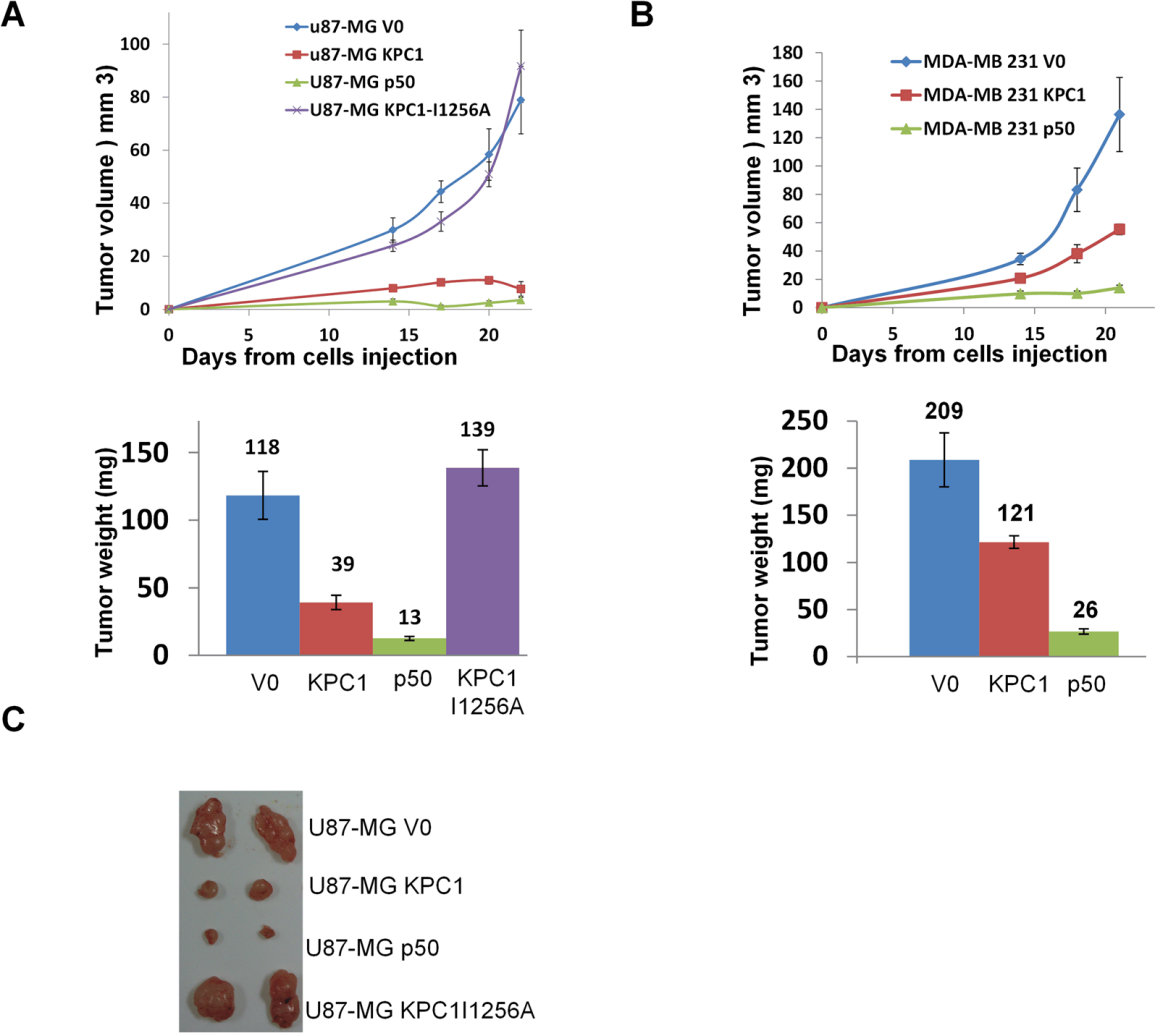
KPC1-mediated generation of p50 from p105, inhibits tumor growth. Growth rates and weights of tumor xenografts grown in mice, and derived from U87-MG (**A**) and MDA-MB 231 (**B**). Cells expressed as indicated, an empty vector (V0), Myc-KPC1, FLAG-p50 or Myc-KPC1I1256A (a RING mutant of KPC1 which is inactive). **C** Tumors derived from U87-MG cells expressing either an empty vector, KPC1, p50 or mutated and inactive KPC1, and harvested 3 weeks after inoculation. Reproduced by permission of Elsevier publisher (license number 5494121109876)

We then tested the effect of overexpressed p50 or KPC1 on model tumors in mice. Xenografts derived from human tumors that stably overexpress KPC1 or p50 and grown in nude mice, showed a significant growth delay compared to control tumors derived from the parental cells that express an empty vector (Fig. 1). Similar to suppressing anchorage independence in cells, the tumor suppressive activity of KPC1 was also dependent on its RING finger domain and on the presence of the 7 amino acids stretch in the ligase to which p105 binds [29, 32].

Based on the identification of the 7 amino acids in KPC1 that bind to p105, it was possible now to design a PROteolysis-TArgeting Chimera, PROTAC. PROTACs are bi-headed molecules where one head binds to the target protein destined for degradation, and the other to a “universal” ligase. The PROTAC brings the target to the appropriate vicinity with the ligase which enables its ubiquitination and subsequent proteasomal activity [33]. The idea was that the 7 amino acids fused to a ubiquitin ligase-binding head (such a Cereblon-CRBN or Von Hippel-Lindau protein-pVHL), will bring p105 to the desired proximity from the ligase, which will result in its ubiquitination and proteasomal processing to p50. We fused the 7 amino acids stretch of KPC1 to a pVHL-binding ligand. The resulted molecule stimulated the ubiquitination of p105 by purified pVHL ubiquitin ligase complex in a cell-free system. In cells, the PROTAC enhanced processing of p105 to p50 and consequently restricted cell growth [32]. It is now important to monitor the effect of the PROTAC on tumor models.

These observations prompted us to uncover the mechanism(s) that underlie the tumor suppressive effect exerted by KPC1 and mediated via its activity on the NF-ĸB pathway. We applied RNA sequencing (RNA-seq) to analyze the gene expression profile of U87-MG tumors expressing either KPC1 or p50. First, we found a strong correlation between gene expression profiles in KPC1- and p50-overexpressing tumors, linking the two proteins to the same pathway (p-value < 10^–300^). Second, the 48 downregulated and 534 upregulated genes were analyzed using a functional analysis database [34]. We found a significant enrichment of different proteins in pathways that are responsible for the remodeling of the tumor microenvironment, among them glycosylated and extracellular matrix proteins, cell migration, cell–cell adhesion, and cell signaling proteins. Finally, analysis of gene annotations revealed a significant increase in the expression of 40 tumor suppressors (p-value < 1.4 × 10^–18^) [29].

Further explaining the suppressive effect, we showed in U87-MG glioblastoma cells and U87-MG-derived xenografts, that excess of p50 downregulates the expression of PD-L1, the major immune checkpoint inhibitor. In p50-expressing tumors, forced expression of PD-L1 under a constitutive promoter, partially impaired tumor suppression [35].

Of note, p65 overexpression in U87-MG or in U87-MG p50-overexpressing cells, upregulates both PD-L1 mRNA and its cognate protein, suggesting that tumorigenic properties of the cells rely partially on the composition of the NF-ĸB dimers—p50 homo-, or p50/p65 hetero-dimers, that initiate anti- or pro-tumorigenic programs, respectively [35]. As expected, overexpression of p65 in p50-overexpressing cells, abrogates partially the suppressive effect of overexpressed p50 [35].

**Fig. 2:**
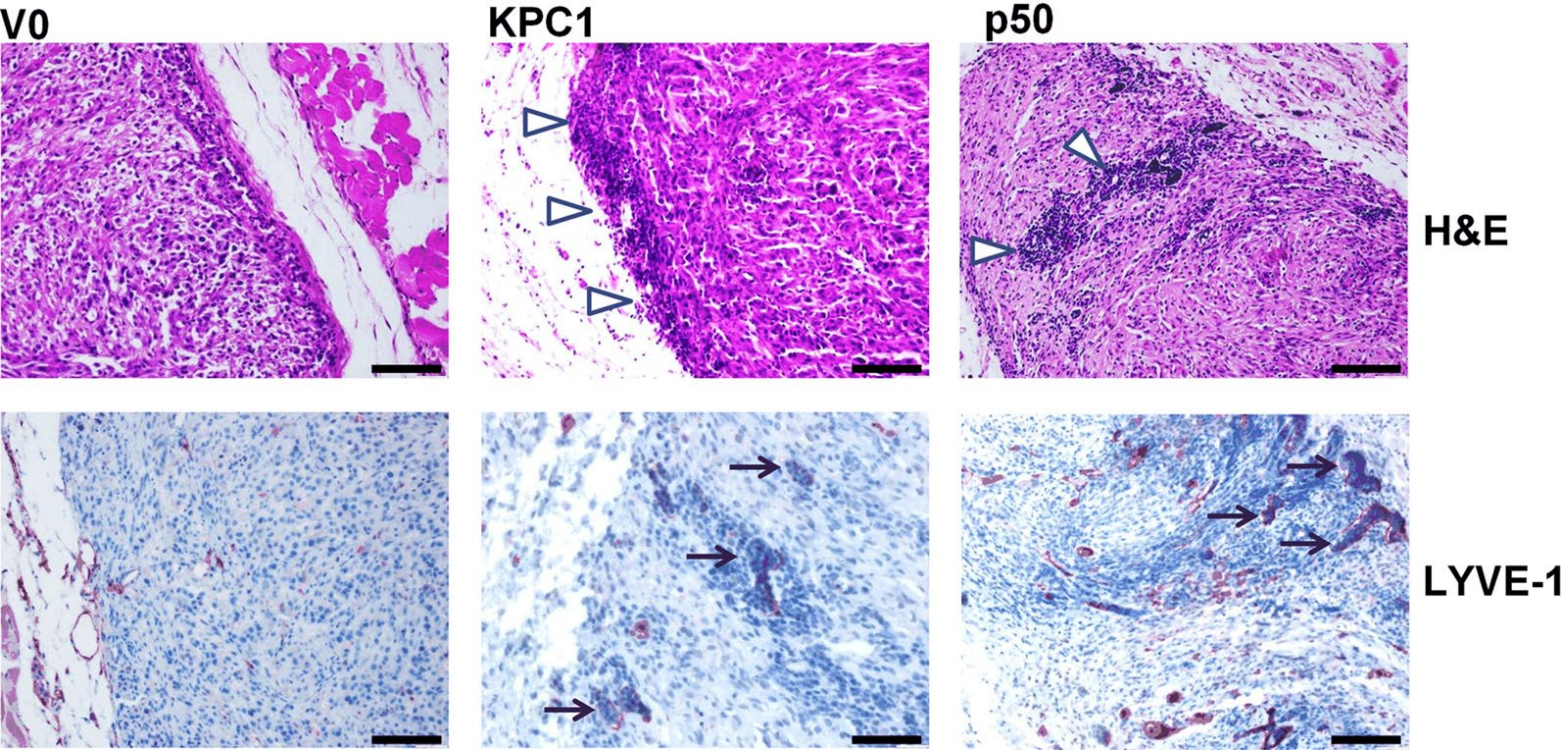
Recruitment of immune cells to xenografts of U87-MG cells overexpressing either KPC1- or p50. U87-MG cells overexpressing KPC1, p50, or a control vector, were injected subcutaneously into nude mice. Tumors were harvested, processed, and stained with hematoxylin and eosin staining (H&E), or immunostained for LYVE-1, a lymphatic vessels marker. Shown are representative photomicrographs of a single tumor from each group. Arrowheads indicate infiltrated immune cells in the KPC1- and p50-expressing tumors. Arrows indicate LYVE-1 immunostaining. Scale bars, 50 μm

The finding that different dimers of NF-ĸB regulate the expression of PD-L1 prompted us to investigate the possible involvement of the immune system in the p50-mediated tumor suppressive mechanism(s). For that, we used hematoxylin–eosin staining of xenografts derived from U87-MG cells expressing V0, KPC1, or p50, and found that they are infiltrated with leukocytes (Fig. 2). To identify these white blood cells, we immunostained the control and p50-overexpressing tumors for different lymphocyte markers. In the suppressed tumors, we observed a strong positive staining for NK cells (NCR1) and macrophages (F4/80) markers. Moreover, taking into consideration the low expression of PD-L1 ligand on the surface of p50-derived tumors, we assumed that NK cells that infiltrate the p50-expressing tumors must be in an active state. Indeed, this assumption was confirmed by positive staining for the NKG2D and Granzyme B markers. On the other hand, we observed negative staining for specific markers of neutrophils (myeloperoxidase) and B cells (B220) [35].

Finally, it was important to demonstrate that the immune system plays an active role in the p50/KPC1-induced tumor suppression. To show that, we used tumors growing in different immune-compromised mouse models. Thus, we compared the size of tumors expressing p50 that grow in nude mice (Hsd:Athymic Nude-*Foxn1*^*nu*^**;** lacking T cells) to that of similar tumors growing in severe combined immunodeficiency mice (SCID, C.B-17/IcrHsd-*Prkdc*^*scid*^; lacking both T and B cells). We could not observe any difference in the size of the tumors growing in the two mice. In contrast, we found a significant difference in the size of similar tumors growing in either nude mice or NOD SCID gamma mice (NSG, NOD.Cg-*Prkdc*^*scid*^* Il2rg*^*tm1Wjl*^/SzJ; lacking T, B, macrophages and NK cells), suggesting the possible involvement of NK cells and macrophages in “attacking” the tumors [35].

To confirm directly the involvement of NK cells and macrophages in the inhibition of tumor growth, we injected them into NSG mice. Both human and mouse NK cells as well as their mixture with macrophages, displayed a significant tumor-restricting effect [35].

At that point, it was important to study the mechanism(s) that attracts the immune cells to the tumors. We suspected that the tumor cells that overexpress p50 or KPC1, secrete cytokines to the circulation which stimulate immune cell migration into the tumors. We found that the pro-inflammatory cytokines CCL3, CCL4, and CCL5 were upregulated in p50-overexpressing tumors. We expressed each of them independently in U87-MG-derived xenografts, and they inhibited tumor growth similar to overexpressed p50. Moreover, we visualized the infiltration of NK cells to U87-MG tumors that overexpress either CCL3, CCL4, or CCL5. These results are in agreement with the infiltration of the same immune cells to p50-expressing tumors [35].

## Conclusion

**Fig. 3:**
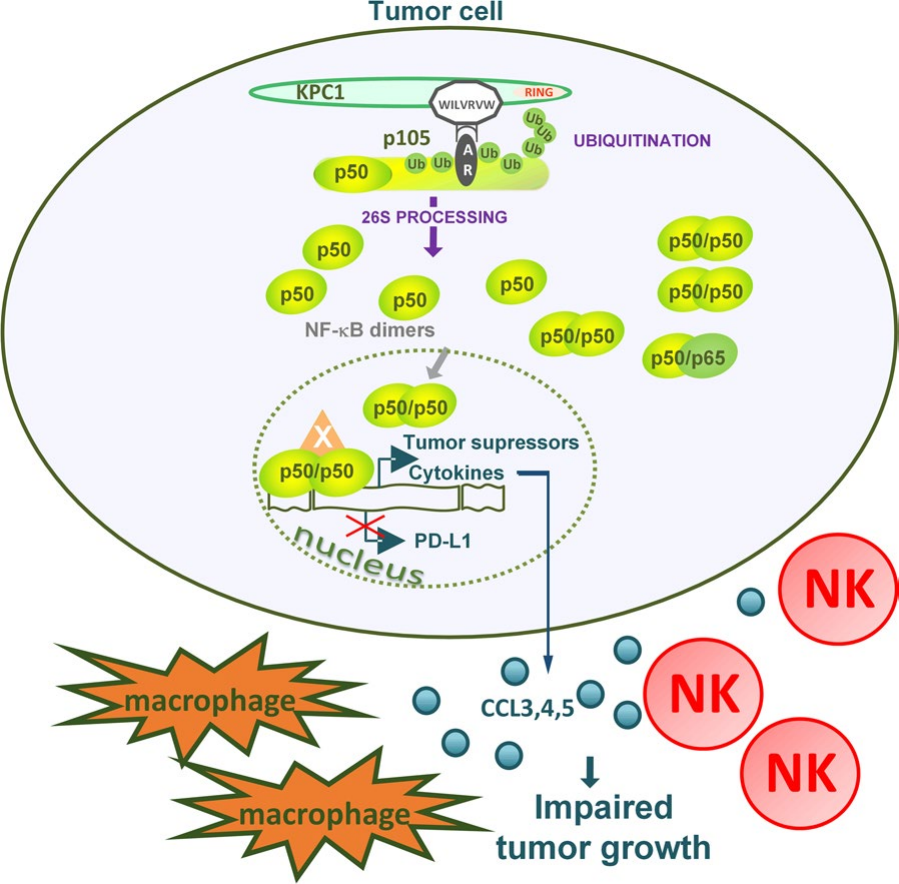
Mechanisms involved in KPC1-dependent impaired tumor growth. KPC1 ubiquitin ligase interacts with the ARs domain of p105 via its 7-aa stretch (WILVRLW). Following ubiquitination, the p105 precursor is processed by the 26S proteasome to generate the p50 active subunit. p50 forms homo- (with p50) or hetero (with p65, the ‘canonical’ NF-κB)-dimers, that move to the nucleus to initiate different transcriptional programs, depending on the composition of the dimers. Excess of p50 probably forms homo-dimers that stimulate: (i) the expression of an array of tumor suppressors and (ii) the expression and secretion of cytokines CCL3, CCL4, and CCL5 that attract the host’s NK cells and macrophages into the tumor, restricting its growth. Also, it inhibits expression of PD-L1 in different tumor cell lines, consequently inhibiting tumor growth

We have shown that p50, when generated in excess via forced processing of its p105 precursor mediated by the ubiquitin ligase KPC1, restricts tumor growth (Fig. 3). This finding clearly points to the fact that a single transcription factor can have different and even opposing activities, dependent on its subunit composition and relative stoichiometry of the different subunits. In this case, NF-κB which typically drives the malignant process, is converted to a tumor suppressor by generation of an excess of p50, one of its subunits, that probably homodimerizes. A similar approach should be taken in analyzing other multi-subunits transcription factors that may unravel, yet unknow, “hidden” novel functions. Importantly, the discovery of the short stretch of amino acids on KPC1 that bind p105, opens the road for the development of a tumor suppressive PROTAC.

## Data Availability

All data in this review were previously published as original articles cited in the References and are therefore available according to the bylaws of the respective periodicals. The data in new Fig. 2 are available as well.

## Abbreviations

NK: Natural killer
siRNA: Small interfering RNA
SCID: Severe combined immunodeficiency
